# Operationalizing risk-appropriate perinatal care in a rural US State: directions for policy and practice

**DOI:** 10.1186/s12913-023-09552-y

**Published:** 2023-06-08

**Authors:** Carly Holman, Annie Glover, Kaitlin Fertaly, Megan Nelson

**Affiliations:** 1 Rural Institute for Inclusive Communities, University of Montana, Missoula, MT USA; 2 School of Public and Community Health Sciences, University of Montana, Missoula, MT USA

**Keywords:** Risk-appropriate care, Perinatal regionalization, Obstetrics, Transport, Rural, LOCATe

## Abstract

**Background:**

Risk-appropriate care improves outcomes by ensuring birthing people and infants receive care at a facility prepared to meet their needs. Perinatal regionalization has particular importance in rural areas where pregnant people might not live in a community with a birthing facility or specialty care. Limited research focuses on operationalizing risk-appropriate care in rural and remote settings. Through the implementation of the Centers for Disease Control and Prevention (CDC) Levels of Care Assessment Tool (LOCATe), this study assessed the system of risk-appropriate perinatal care in Montana.

**Methods:**

Primary data was collected from Montana birthing facilities that participated in the CDC LOCATe version 9.2 (collected July 2021 – October 2021). Secondary data included 2021 Montana birth records. All birthing facilities in Montana received an invitation to complete LOCATe. LOCATe collects information on facility staffing, service delivery, drills, and facility-level statistics. We added additional questions on transport.

**Results:**

Nearly all (96%) birthing facilities in Montana completed LOCATe (*N* = 25). The CDC applied its LOCATe algorithm to assign each facility with a level of care that aligns directly with guidelines published by the American Academy of Pediatrics (AAP) and the American College of Obstetricians and Gynecologists (ACOG), and Society for Maternal-Fetal Medicine (SMFM). LOCATe-assessed levels for neonatal care ranged from Level I to Level III. Most (68%) facilities LOCATe-assessed at Level I or lower for maternal care. Close to half (40%) self-reported a higher-level of maternal care than their LOCATe-assessed level, indicating that many facilities believe they have greater capacity than outlined in their LOCATe-assessed level. The most common ACOG/SMFM requirements contributing to the maternal care discrepancies were the lack of obstetric ultrasound services and a physician anesthesiologist.

**Conclusions:**

The Montana LOCATe results can drive broader conversations on the staffing and service requirements necessary to provide high-quality obstetric care in low-volume rural hospitals. Montana hospitals often rely on Certified Registered Nurse Anesthetists (CRNA) for anesthesia services and telemedicine to access specialty providers. Integrating a rural health perspective into the national guidelines could enhance the utility of LOCATe to support state strategies to improve the provision of risk-appropriate care.

## Background

After steady increases over the last 25 years, the United States has the highest maternal mortality rate among developed nations [1, 2]. Inadequate and fragmented care prevents pregnant patients from getting timely, risk-appropriate care, exacerbating the consequences of dangerous obstetric complications [3]. Rural populations face additional barriers, including distance to care, hospital and obstetric unit closures, and other social determinants of health, that further increase their risk for poor obstetric outcomes, including severe maternal morbidity (SMM) [4, 5]. The alarming maternal health outcomes in the United States, especially in rural communities, underscore the urgency of improving the delivery of perinatal care through targeted system-level improvements [5, 6].

Geography is a significant factor that determines the location where a patient delivers [7]. Maintaining obstetric services in small rural hospitals creates challenges due to low birth volume, workforce issues, and meeting the cost demands [8, 9]. As more rural obstetric units close, distance to care becomes a greater burden for rural birthing people [10]. When hospitals or obstetric units close, more patients go without prenatal care and use the emergency room for obstetric services [11–13]. Perinatal regionalization, which emphasizes right-time and right-place care provision through referral networks, has particular importance in rural settings where pregnant people might not live in a community with a birthing facility or specialty care [4, 5]. Pregnant people living in rural states can benefit from specialized approaches that target the specific challenges faced by geographically remote communities and demographically sparse populations [3, 14].

Montana’s extreme rurality presents a useful case study for assessing systems of risk-appropriate care that can inform similarly positioned populations. Montana’s health system spans 56 mostly rural counties and seven remote Indian Reservations. Montana has 51 counties classified as rural (micropolitan/non-core) [15]. Less than half (45%) of reproductive age people in Montana live within 50 miles of critical perinatal care services compared to 94% of the reproductive age population in the United States [3]. In 2004, 49% of counties in Montana had a hospital with an obstetric unit [16], which has decreased to 40% in 2022 [17]. Inequities in obstetric care access and health outcomes persist in the state [18, 19]. American Indian Alaska Native (AIAN) birthing people are 20 times more likely to give birth at a hospital that does not have an obstetric unit and travel significantly farther to access obstetric care, even compared to White birthing people in rural areas [18]. In a recent study on SMM in Montana, race and the rurality of a birthing person’s county were both associated with an increased risk of SMM [19]. Efforts to improve care must account for the supply of obstetric services and the availability of specialty care across the population [10, 18].

The March of Dimes first put forward risk-appropriate care as a strategy for improving maternal and neonatal health in the landmark 1975 report, *Toward Improving the Outcome of Pregnancy* [20]. States responded to the guidance and began implementing the recommended regional systems of care to ensure high-risk pregnant people and infants receive care at a facility with the staff and resources to meet their needs [20, 21]. Regionalization contributes to improved birth outcomes by standardizing the management of high-risk cases and ensuring care occurs at an appropriate facility [22]. A meta-analysis looking at associations between hospital level at birth and neonatal or predischarge mortality showed that high-risk infants (very low birth weight and very pre-term) born at lower-level facilities < Level III experienced increased likelihood of neonatal or predischarge death [23]. In 2012, the American Academy of Pediatrics (AAP) published updated recommendations that included defined levels of care based on specific facility capabilities [24]. While perinatal regionalization intended to include maternal and neonatal care, early efforts focused primarily on the neonate [3, 21]. To combat the high rates of maternal morbidity and mortality in the United States, the American College of Obstetricians and Gynecologists (ACOG) and the Society for Maternal–Fetal Medicine (SMFM) published the 2015 Levels of Maternal Care Obstetric Care Consensus, which defined maternal levels of care distinct from the neonatal levels of care [25]. ACOG/SMFM released an update to these guidelines in 2019 [6]. A study of nine maternal mortality review committees (MMRC) identified “adopting levels of maternal care/ensure an appropriate level of care determination” as a common recommendation to prevent maternal deaths [26]. In response to state variations in classifying levels of care, the Centers for Disease Control and Prevention (CDC) developed the Levels of Care Assessment Tool (LOCATe) as a nationally standardized assessment that maintains alignment with AAP [24] and ACOG/SMFM [6] guidelines [21].

Though perinatal regionalization has gained significant momentum as a strategy to improve birth outcomes [14], little information exists on how these systems function in rural and remote settings. Our study of the risk-appropriate care landscape in Montana’s perinatal health system seeks to fill this critical knowledge gap. Through the implementation of the CDC LOCATe, this study aimed to 1) describe the statewide perinatal care system through levels of care, 2) compare the current perinatal care system to the AAP and ACOG/SMFM levels of care, and 3) understand how risk-appropriate care is operationalized in a rural setting to identify implications for practice, policy, and future research to support improved perinatal care outcomes.

## Methods

### Participants

All birthing facilities (hospitals with an obstetric unit) (*N* = 26) in Montana received an invitation to participate in the LOCATe initiative. We sent a letter to the Administrator, Director of Nursing, and Quality Improvement Coordinator introducing the Montana LOCATe Initiative and requesting the facility identify a LOCATe champion. The LOCATe champion, most often a nurse, attended a webinar on completing the assessment. The LOCATe champion engaged others at the facility to gather the necessary information including nurse managers, physicians, data managers, and quality coordinators, and submitted the assessment on behalf of the facility.

### Instrument

We implemented version 9.2 of the CDC LOCATe assessment. The LOCATe assessment has a section on maternal care and neonatal care. LOCATe collects information on facility staffing and availability, type and volume of services, transport, drills and protocols, and facility-level statistics. LOCATe also asks facilities to self-report their level of neonatal and maternal care based on the AAP and ACOG/SMFM guidelines [21]. We created and added a module to the LOCATe assessment to collect additional information on training, accessibility, equipment/technology, and blood products as part of a broader maternal health system needs assessment. We also included a module with supplementary questions on transport that the CDC developed in partnership with another rural state. We modified the questions based on input from rural obstetric clinical experts.

### Survey administration

The data collection occurred from July 23, 2021, to October 31, 2021. LOCATe champions received an email with instructions and the online survey link. We administered the assessment through the REDCap platform. The survey remained open for 14 weeks, and participants received email reminders and follow-up calls throughout the recruitment period. After receiving all completed assessments, we sent the deidentified data to CDC for analysis. CDC provided us with preliminary results, and we completed a data validation process with all the facilities. This process occurred from November 15, 2021, to December 17, 2021. As part of the data validation process, facilities reviewed their preliminary results, confirmed data accuracy, or made updates based on clarification of questions. Upon completion, we sent the deidentified data back to CDC for final analysis. The University of Montana Institutional Review Board (IRB) determined this study did not qualify as human subjects research.

### Data analysis

CDC developed an algorithm for assessing the maternal and neonatal level of care. States and jurisdictions participating in LOCATe establish a Memorandum of Understanding (MOU) with CDC regarding data management and analysis. The algorithm utilizes a scoring system for each of the questions that refer directly to staffing and service specifications in the AAP and ACOG/SMFM guidelines. Each question is scored with equal weight, and an overall maternal and neonatal level of care is provided for each participating facility [21]. AAP and ACOG/SMFM include four levels of care (Level I – Level IV). The CDC LOCATe algorithm includes a fifth level < Level I which is assigned to facilities that do not meet the minimum requirements for Level I. Upon completion of the analysis, the CDC sent the final analytic files of the Montana LOCATe data. We conducted additional analyses looking at driving distance from lower-level facilities (< Level I and Level I) to the nearest higher-level facility (Level II, Level III, and Level IV). Driving distance was calculated in miles utilizing google maps. This study also includes an analysis of the number of births by the level of maternal and neonatal care, using the LOCATe data and Montana Vital Records for all births in 2021 [27]. We conducted all analyses of the additional survey module using STATA 17.

## Results

Twenty-five (96%) of the birthing facilities in the state participated in LOCATe. Table 1 shows the levels of neonatal care, including self-reported and LOCATe-assessed levels. As shown in Table 1, about half of facilities LOCATe-assessed at Level I (48%). Montana does not have any birthing facilities that meet the requirements of Level IV neonatal care. Several facilities (12%) LOCATe-assessed higher than their self-report by one level.

**Table 1:** Self-Reported and LOCATe-Assessed Levels of Neonatal Care in Montana Birthing Facilities

Facility (*N* = 25)	Self Report n (%)	LOCATe Assessment n (%)
Level I	15 (60.0)	12 (48.0)
Level II	5 (20.0)	8 (32.0)
Level III	5 (20.0)	5 (20.0)
Level IV	0	0

Table 2 outlines the levels of maternal care, including self-reported and LOCATe-assessed levels. Most facilities (68%) LOCATe-assessed at Level I or lower for maternal care. Close to half (40%) had a higher self-reported level than their LOCATe-assessed level. The most common ACOG/SMFM requirements contributing to the level of maternal care discrepancies were the lack of obstetric ultrasound services and a physician anesthesiologist. Level I maternal care requires a hospital have limited or standard obstetric ultrasound with interpretation services readily available at all times. Level I maternal care anesthesiology requirements can be met by either a certified registered nurse anesthetist (CRNA) or anesthesiologist physician. Level II requires a physician anesthesiologist to be readily available at all times, and Level III and IV require a physician anesthesiologist that is also board-certified with fellowship training or experience in obstetric anesthesia.

**Table 2:** Self-Reported and LOCATe-Assessed Levels of Maternal Care in Montana Birthing Facilities

Facility (*N* = 25)	Self Report^a^ n (%)	LOCATe Assessment n (%)
< Level I	N/A	6 (24.0)
Level I	13 (56.5)	11 (44.0)
Level II	5 (21.7)	6 (24.0)
Level III	4 (17.4)	1 (4.0)
Level IV	1 (4.3)	1 (4.0)

^a^2 facilities responded ‘unknown’ in self-report; *N* = 23

Births occurred at facilities across all levels of maternal and neonatal care. Most births (72%) occurred at facilities that LOCATe-assessed at Level II or lower for maternal care. Table 3 includes births by LOCATe-assessed level and those that occurred at birth centers, home births, and other hospital births. Table 3 shows where the birth happened and does not account for where the obstetric care might have started and the transfer of care.

**Table 3:** Montana Births by Facility Type and LOCATe-Assessed Level of Neonatal Care and Maternal Care, 2021 *N* = 11,247^a^

Facility Type	Births n (%)	
	Level of Neonatal Care	Level of Maternal Care
< Level I	N/A	708 (6.3)
Level I	1435 (12.8)	1838 (16.3)
Level II	3561 (31.7)	5514 (49.0)
Level III & Level IV	5613 (49.9)	2549 (22.7)
Births occurring outside a LOCATe-Assessed Facility		
Other hospital births^b^	141 (1.3)	
Birth centers^c^	171 (1.5)	
Home births	326 (2.9)	

^a^Montana Vital Records

^b^Births occurring in a birthing hospital that does not have a LOCATe-assessed level of care and/or non-birthing hospitals

^c^Accredited Birth Centers (free-standing facilities that are not hospitals) provide care for low-risk women with uncomplicated singleton term vertex pregnancies who are expected to have an uncomplicated birth

Table 4 provides the calculated driving distance from < Level I and Level I (basic care) maternal care facilities to Level II (specialty care), Level III (subspecialty care), and Level IV (regional perinatal care health centers) in Montana. Table 4 also includes the driving distance from Level I (well-born nursery) neonatal care facilities to Level II (special care nursery) and Level III (NICU). These results do not include driving distance to out-of-state hospitals. Sometimes, the closest higher-level facility is located in a neighboring state. The lower-level (< Level I and Level I) maternal care facilities are at a median distance of about 100 miles from the nearest Level II facility and around 300 miles from a Level III or IV hospital. Level I neonatal facilities are also far from higher care levels, at a median distance of 78 miles from a Level II and 107 miles from a Level III hospital. Montana has no Level IV neonatal care facilities, requiring transport to an out of state hospital to receive these services. The closest Level IV NICUs are in Idaho, Washington, Utah, and Colorado.

**Table 4:** Calculated Driving Distance in Miles to Nearest Higher-Level Facility

Level of Care	Driving distance in miles to nearest higher-level facility in Montana, Median [IQR]*		
**Maternal Level of Care**	**Level II**	**Level III**	**Level IV**
< Level I (*n* = 6)	96.5 [54–273]	325.5 [145–412]	326 [145–412]
Level I (*n* = 11)	90 [57–119]	257 [227–399]	258 [227–399]
**Neonatal Level of Care**	**Level II**	**Level III**	**Level IV**
Level I (*n* = 12)	78 [60.5–130]	107 [67.5–199.5]	N/A

^*^ *IQR* Interquartile range

As illustrated in Table 5, about a quarter (28%) of Montana birthing facilities receive neonatal transports. Of the facilities that reported receiving neonatal transports, two LOCATe-assessed at Level II neonatal care and five at Level III neonatal care.

**Table 5:** Neonatal Transport by Level of Neonatal Care in Montana Birthing Facilities

Level of Neonatal Care	Receive any neonatal transports n (%)	Receive complicated high-risk neonates n (%)	Receive convalescent neonates n (%)
Montana overall (*N* = 25)	7 (28.0)	6 (85.7)	5 (71.4)
LOCATe-assessed Level I (*n* = 12)	0	0	0
LOCATe-assessed Level II (*n* = 8)	2 (25.0)	1 (50.0)	2 (100.0)
LOCATe-assessed Level III (*n* = 5)	5 (100.0)	5 (100.0)	3 (60.0)

As illustrated in Table 6, 56% of facilities reported having a written plan for the transport of complicated obstetric patients. About a quarter (21%) stated their transport plan includes a mechanism to facilitate and openly accept maternal transports from lower-level facilities. Nearly half (44%) of facilities do not have a written plan for the transport of complicated obstetric patients. Many of these facilities reported having a general transport plan but not one specific to obstetric patients.

**Table 6:** Maternal Transport by Level of Maternal Care in Montana Birthing Facilities

Level of Maternal Care	Written plan for transport of complicated obstetric patients (any) n (%)	Plan includes mechanism for maternal transport to higher-level facility available at all times n (%)	Plan includes mechanism to facilitate and openly accept maternal transports from lower-level facilitiesn (%)
Montana overall (*N* = 25)	14 (56.0)	13 (92.9)	3 (21.4)
LOCATe-assessed < Level I (*n* = 6)	2 (33.3)	2 (100.0)	0
LOCATe-assessed Level I (*n* = 11)	7 (63.6)	7 (100.0)	0
LOCATe-assessed Level II (*n* = 6)	3 (50.0)	2 (66.7)	1 (33.3)
LOCATe-assessed Level III (*n* = 1)	1 (100.0)	1 (100.0)	1 (100.0)
LOCATe-assessed Level IV (*n* = 1)	1 (100.0)	1 (100.0)	1 (100.0)

The additional questions added to LOCATe gathered further details on the transport process. About half (52%) of facilities have a protocol describing under what circumstances a transport should be arranged and have a specific contact to call and arrange transfer with the receiving facility. A quarter (28%) of facilities reported having a written transport agreement with another hospital. Of those with a transport agreement, few included details about processes for follow-up communication (12%) and back transport (16%).

## Discussion

An essential component of risk-appropriate care involves an accurate and shared understanding of facility capabilities and level of care [6, 28]. Three facilities (12%) had a discrepancy between their self-reported level of neonatal care and their LOCATe assessed level. All facilities LOCATe-assessed higher by one level. In a national study of health facilities that implemented LOCATe, of the 721 hospitals that self-reported a level of neonatal care, 33% had discrepancies between their self-reported and LOCATe-assessed levels [29]. A quarter (25%) of these facilities LOCATe assessed higher than their self-report [29]. Close to half (40%) of facilities self-reported a higher-level of maternal care than their LOCATe-assessed level. Montana’s results align with national trends, with 41% of facilities self-reporting higher than the LOCATe-assessed level of maternal care [28]. The most common ACOG/SMFM requirements contributing to the maternal care discrepancies in Montana aligned with national results, including service and staffing requirements (obstetric ultrasound and physician anesthesiologist) [28]. A coordinated system relies on a shared understanding of the levels of care both within and between facilities [22]. Discrepancies might reflect a lack of familiarity with the level of care guidelines [28, 29]. Facilities make decisions regarding the type of patients they can treat based on their perceived capacity. Inaccurate self-assessment can lead to poor outcomes if facilities treat or accept patients outside their capabilities [26, 28, 29]. It can also impact decisions on transport and back-transport, impacting patients’ ability to receive care in their communities. Montana’s results point toward a need to strengthen facilities’ understanding of levels of care, standardizing the implementation of risk-appropriate care across the health system.

Systems of care operate differently in rural and urban areas due to the geographic characteristics and volume of services [14]. The LOCATe assessment provided valuable information on the geographic distribution of perinatal care in Montana. The median driving distance from a maternal <Level I and Level I facility to the nearest Level II facility requires traveling close to 100 miles. Several facilities in Montana met all of the Level II or Level III maternal care requirements, aside from the physician anesthesiologist. CRNAs serve as the predominant anesthesia providers in rural communities and fill a vital role by ensuring communities have access to anesthesia services [30]. Many states, including Montana have less restrictive policies allowing CRNAs to practice without physician supervision, contributing to a greater supply of anesthesia workforce in rural communities [30]. CRNAs are licensed as independent practitioners in Montana and operate as autonomous healthcare professionals [31]. While several facilities in Montana did not meet the ACOG/SMFM anesthesia physician staffing requirements, they could still provide obstetric anesthesia care with CRNAs. Another area contributing to maternal care discrepancies included the < Level I facilities. A quarter (24%) of hospitals in Montana LOCATe-assessed at < Level I; these facilities are Critical Access Hospitals (CAHs) with low birth volumes. CAHs serve a vital role in rural health systems by increasing access to obstetric services in remote communities. Obstetric ultrasound availability accounted for the most common reason these facilities did not meet the ACOG/SMFM Level I requirements. These hospitals had the necessary equipment and staff but did not offer services 24/7.

The Montana results can drive broader conversations on the staffing and service requirements necessary to provide high-quality obstetric care in low-volume rural hospitals. Integrating a rural health perspective into the AAP and ACOG/SMFM guidelines could enhance the utility of LOCATe to support state strategies to improve the provision of risk-appropriate care [28]. The Montana LOCATe results bring forth important questions regarding the application of the ACOG/SMFM levels of care in low-volume rural hospitals. Should the < Level I facilities in Montana work toward meeting Level I requirements by expanding the availability of obstetric ultrasound? Or do their current services meet patient demand, in which case, could ACOG/SMFM include a low-volume/rural Level I classification that accounts for rural–urban differences? Including a rural designation criteria would also emphasize the value of rural facilities in expanding access to obstetric care during a time with many rural hospital and obstetric unit closures [10].

A well-functioning system of risk-appropriate care relies on solid transport protocols to ensure that at-risk maternal and neonatal patients receive care at a facility prepared to meet their needs [32, 33]. Results from the Montana LOCATe assessment suggest that enhancing the provision of risk-appropriate care in the state will require improvements to the transport system. Close to half (44%) of facilities that participated in LOCATe do not have a maternal-specific transport plan. About a quarter (28%) reported having a written transport agreement with another hospital. Few included details on follow-up communication (12%) and back transport (16%). A study of Ohio MMRC data found that improvements in the maternal transport system can prevent maternal deaths through earlier transfer to a higher-level of care [33]. ACOG/SMFM recommends Level I and Level II facilities partner with higher-level facilities to establish a maternal transport plan and agreement to address patient needs when complications arise [6]. ACOG/SMFM detail components of a transport plan including, a standardized process for risk assessment and determination of conditions requiring transport, a procedure for facilitating transport, and a comprehensive communication system between hospitals, providers, and transport teams [6]. Though all perinatal care systems rely on transport, its importance in rural communities cannot be overstated. Distance to a higher-level facility, harsh winters, and communities with limited obstetric care further complicates arranging timely transport, requiring additional planning and preparedness [33]. Rural health systems need to plan for instances when transport cannot occur, necessitating the development of distinct strategies within regions and across the state [3, 33]. Strategies might include telemedicine protocols with higher-level facilities and coordination with facilities in neighboring states that may be the closest specialty care [3, 33].

The LOCATe assessment produced important information on perinatal care capacity across Montana; however, it did not provide a complete picture. Due to distance to care, birthing people might seek care or deliver at a hospital without an obstetrics unit [13]. Montana has 34 CAHs that do not have an obstetric unit. These CAHs fill gaps in the system by providing obstetric services in communities without a birthing facility. In rural health systems, the uneven distribution of birthing facilities and vast geographic areas necessitates regionalization strategies that include all available perinatal care [10]. During the LOCATe implementation process, Montana surveyed hospitals without an obstetric unit on emergency obstetrics services to gather a comprehensive picture of perinatal care capacity in the state. There may be value in expanding the LOCATe assessment to include an accompanying emergency obstetrics services module for implementation with non-birthing facilities in rural states. This information, accompanied by the LOCATe results can guide states to build intentional partnerships across hospitals to improve risk-appropriate care and ensure safety for emergency births.

There are several limitations to this study. One individual served as the LOCATe champion and completed the assessment on behalf of the facility. If the LOCATe champion did not engage other obstetric team members, the responses could reflect their knowledge of the facility’s services and might not be comprehensive. LOCATe includes a set of detailed instructions and definitions. Respondents that do not thoroughly read these instructions might answer the questions based on their own interpretation leading to incorrect information. Version 9.2 of the LOCATe assessment does not include an option to self-report as < Level I maternal care. Some maternal level of care discrepancies might be due to facilities not being able to self-report as < Level I.

## Conclusions

While perinatal regionalization has led to improved outcomes for birthing people and infants both domestically and internationally, the structure and strategies that work in one system might not meet the needs of another [14]. The Montana LOCATe results have identified important implications for policy and practice to support the development of regionalized perinatal care in rural states. Rural health systems must prioritize formalizing relationships among facilities, leverage telemedicine services to expand access, and strengthen transport systems to address the challenges of distance to care. Adequately staffed and equipped facilities at each level of care and regionalized relationships increase the ability of birthing people to deliver in their communities while providing support for complex and emergent situations [6]—ultimately shaping a maternal health system that meets the unique needs of the population.

## Data Availability

The data that support the findings of this study are available on request from the corresponding author [CH]. The data are not publicly available due to them containing information that could compromise participating hospitals’ privacy. If other researchers want to access the Montana LOCATe dataset, a DUA will be established with the University of Montana to uphold the commitment of confidentiality that was made with participating hospitals.

## Abbreviations

AAP: American Academy of Pediatrics
ACOG: American College of Obstetricians and Gynecologists
AIAN: American Indian Alaska Native
CAH: Critical Access Hospital
CDC: Centers for Disease Control and Prevention
CRNA: Certified Registered Nurse Anesthetist
LOCATe: CDC Levels of Care Assessment Tool
MFM: Maternal–Fetal Medicine Specialist
SMFM: Society for Maternal-Fetal Medicine
SMM: Severe Maternal Morbidity
MMRC: Maternal Mortality Review Committee
MOU: Memorandum of Understanding
